# Investigation into a national outbreak of STEC O157:H7 associated with frozen beef burgers, UK, 2017

**DOI:** 10.1017/S0950268820001582

**Published:** 2020-07-16

**Authors:** Lisa Byrne, Lukeki Kaindama, Maria Bentley, Claire Jenkins, Heather Aird, Isabel Oliver, Karthik Paranthaman

**Affiliations:** 1 Gastrointestinal Infections Department, National Infections Service, Public Health England, 61 Colindale Avenue, London, NW9 5EQ, UK; 2 Environmental Health, Hambleton District Council, Civic Centre, Stone Cross, Northallerton, North Yorkshire, DL6 2UU, UK; 3 Gastrointestinal Bacteria Reference Unit, National Infections Service, Public Health England, 61 Colindale Avenue, London, NW9 5EQ, UK; 4 Food, Water and Environmental Laboratory, Public Health England, Block 10, NAFIC, Sand Hutton, York, YO41 ILZ, UK; 5 Field Service, National Infections Service, Public Health England, 2 Rivergate, Bristol, UK

**Keywords:** Food-borne infections, gastrointestinal infections, outbreaks, Shiga-like toxin-producing *E. coli*

## Abstract

In November 2017, Public Health England identified an outbreak of Shiga toxin-producing *Escherichia coli* O157:H7 in England where whole genome sequencing results indicated cases were likely to be linked to a common source, and began investigations. Hypothesis generation included a review of enhanced surveillance data, a case-case study and trawling interviews. The hypothesis of interest was tested through the administration of focussed questionnaires and review of shopping history using loyalty card data. Twelve outbreak cases were detected, eight were hospitalised and four developed haemolytic uraemic syndrome. Frozen beef burgers supplied by a national retailer were identified as the vehicle of the outbreak. Testing of two left-over burger samples obtained from the freezers of two separate (unlinked) cases and a retained sample from the production premises were tested and found to be positive for the outbreak strain. A voluntary recall of the burgers was implemented by the retailer. Investigations at the production premises identified no contraventions of food safety legislation. Cooking guidance on the product packaging was deemed to be adequate and interviews with the cases/carers who prepared the burgers revealed no deficiencies in cooking practices at home. Given the long-shelf life of frozen burgers, the product recall likely prevented more cases.

## Introduction

Shiga toxin-producing *Escherichia coli* (STEC) O157:H7 is a zoonotic pathogen that can cause gastrointestinal illness. Around 700 cases of STEC O157:H7 are reported annually in England [1]. STEC O157:H7 are of public health concern due to the potential severity of disease, ranging from mild to more severe bloody diarrhoea. Haemolytic uraemic syndrome (HUS), a severe multisystem disorder, can develop as a complication in 5–15% of cases and the risk is dependent on the age and sex of the case, and the pathogenic characteristics of the strain of STEC [2–4].

The natural reservoir of STEC O157:H7 is the gastrointestinal tract of ruminant animals, predominantly cattle and sheep in the UK. Human infection can occur through direct or indirect contact with animals or their environment, or consumption of contaminated food or water. Infections can be sporadic or comprise outbreaks. Foodborne outbreaks of STEC O157:H7 in England have been associated with contaminated raw or undercooked meat, or cooked meats which had been cross-contaminated; raw milk and raw milk products and contaminated raw vegetables and salads [5]. Measures to prevent infection from contaminated food include adequate cooking of meat products before consumption and avoiding cross-contamination of ready to eat products from raw meat. The relative importance of different vehicles in causing outbreaks in England has changed over time; meat-related outbreaks of STEC are less frequently detected since significant meat hygiene practices were implemented in the late 1990s [5]. Conversely, detection and investigation of outbreaks associated with fresh produce have increased in regularity both in the UK [6–11] and the USA [12].

Since 2015, whole genome sequencing (WGS) of all STEC isolates in the UK has been undertaken to provide highly discriminatory typing for public health surveillance and to facilitate outbreak detection and investigation. Isolates within five single nucleotide polymorphisms (SNPs) of each other are likely to have arisen from the same source and/or vehicle [13, 14].

On 13 November 2017, Public Health England (PHE) identified a suspected outbreak of STEC O157:H7 through routine surveillance, when four cases with STEC O157:H7 isolates with the same phage type (PT2) and within five SNPs of each other were identified. We present key findings from subsequent investigations and control measures undertaken in response to this outbreak.

## Methods

### Clinical microbiology

Faecal specimens from patients were processed in local hospital microbiology laboratories for identification of *Salmonella*, *Campylobacter*, *Shigella* spp. and STEC O157:H7. Presumptive isolates of STEC O157:H7 were sent to the PHE Gastrointestinal Bacteria Reference Unit (GBRU) for confirmation, identification of phage type (PT) and the presence of Shiga toxin (*stx*) genes by PCR [15]. WGS was undertaken as described previously [16].

### Outbreak case definition

A confirmed case was a case of STEC O157:H7 belonging to the SNP designation 9.148.298.681.4005.4232.% with an onset date on or after 28 September 2017.

### Epidemiological investigations

Prospective and retrospective case ascertainment was undertaken by reviewing PT and WGS data for STEC cases reported in 2017. The UK posted the outbreak on the Epidemic Intelligence Information System sharing the WGS accession numbers to ascertain whether related cases had been seen elsewhere.

In England, PHE has operated a National Enhanced Surveillance System for STEC (NESSS) since 2009, described in detail previously [17]. For every laboratory-confirmed case of STEC O157, a detailed history is obtained for the 7 days prior to onset of illness using an enhanced surveillance questionnaire (ESQ). A similar process is in place in Scotland.

Hypothesis generation comprised three elements: review of ESQ data on outbreak cases, a case-case analysis and further interviews using a trawling questionnaire. A case-case study was undertaken to compare exposures among the 11 outbreak cases in England in NESSS against 537 primary non-outbreak cases (control group) who were not associated with travel abroad or any other known outbreaks. Non-outbreak cases were matched to outbreak cases on gender and age group and selected as those with symptom onset between 1 September and 31 December in the years 2009 through to 2017. Only food exposures were examined as descriptive epidemiology excluded foreign travel or animal exposures. To assess differences in exposures for outbreak and non-outbreak cases, univariable analysis was undertaken through the calculation of odds ratios (ORs). Exposures not collected as standard binary questions, including shopping at national retailers, were coded through parsing free text fields into coded variables. Data handling and analysis was undertaken in Stata v12.0 (Stata Corp, Texas).

Outbreak cases were re-interviewed using a trawling questionnaire to collect a more detailed food history. Following hypothesis generation, cases were again re-interviewed using a focused questionnaire and asked specifically about their consumption or handling of the suspected food products, including product names, brands, purchased from where, whether products were fresh or frozen, pack size and batch code. Website links to the UK major supermarkets relevant products containing pictures were provided as an *aide memoire* to cases. Supermarket loyalty card details were requested from cases and were provided to the retailers for them to compare and review food purchase history of cases, as recorded on their databases. Consent for sampling any left-over product was requested at this stage.

### Food chain investigations

The Food Standards Agency (FSA) worked with the local authority (LA) to investigate the supply chain and assess compliance with food safety regulations. The designated food safety regulator undertook a formal inspection of the plant, from intake of the ingredients to dispatch of the final retail product. A review of the hazard analysis and critical control point document, cleaning records and production records was undertaken. The company's internal sampling procedure and results were examined.

Verification environmental sampling was undertaken at the plant to assess the effectiveness of cleaning at the production site. Formal food samples, including raw meat and final product, were submitted to the PHE Food, Water and Environmental (FW&E) laboratory. Where available, samples of potential food source/vehicles were obtained from the home of cases and tested at the FW&E laboratories. All food samples were collected and transported to the FW&E laboratories in cool boxes using standard PHE procedures.

The FW&E laboratory tested all food and environmental samples using PHE Standard Method F17 based on BS EN ISO 16654:2001 Detection of *Escherichia coli* O157 by Automated Immunomagnetic Separation. In addition, retained food samples from the freezers of cases were also tested using PHE Standard method M6 based on EN ISO/TS 13136:2012 Microbiology of food and animal feed – real-time polymerase chain reaction (PCR)-based method for the detection of food-borne pathogens – Horizontal method for the detection of STEC and the determination of O157, O111, O26, O103 and O145 serogroups. PHE Standard method F13 based on BS EN ISO 6579-1:2017 Horizontal method for the detection, enumeration and serotyping of *Salmonella* and PHE Standard method F8 based on BS ISO 16649-2:2001 – Horizontal method for the enumeration of *β*-glucuronidase-positive *E. coli* – Part 2: Colony-count technique at 44 °C using 5-bromo-4-chloro-3-indoyl *β*-D-glucuronide were also used when testing samples collected from the meat preparation premises. Isolates of STEC O157:H7 from food samples were referred to GBRU for further characterisation and WGS as per clinical isolates.

### Further investigations

Following confirmation of the food vehicle, cases were interviewed again with a specific questionnaire to examine food storage and handling practices within the home. The FSA reviewed the adequacy of cooking instructions provided on the product pack and the retailer undertook additional laboratory tests on the cooking instructions.

## Results

### Microbiological

All 12 cases in the outbreak were identified as STEC O157:H7 PT2 harbouring *stx* subtype, *stx2a*. WGS confirmed that isolates from nine cases were identical and the remaining three isolates differed 1–2 SNPs from the outbreak profile.

The outbreak strain was unique and had not been detected in the preceding 3 years of routine WGS for STEC isolates in England. The outbreak strain fell within the same clade as STEC O157:H7 isolated from UK livestock indicating that the source was of UK domestic origin.

### Epidemiological investigations

**Fig. 1.**
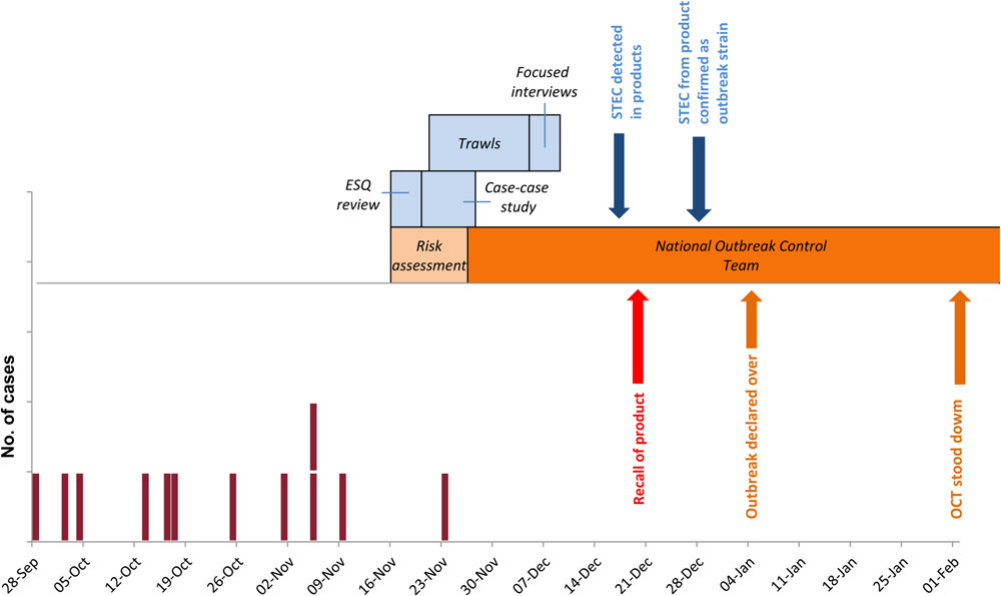
Epidemic curve for outbreak cases and timeline of key events in the investigation.

Cases had onset dates between 28 September 2017 and 23 November 2017 (Fig. 1).

Eleven cases were resident in England and were distributed nationally and one case was resident in Scotland. Nine cases were male and three were female. Cases ranged in age from 1 to 65 years with a median of 16 years. Information on clinical symptoms was available for the 11 cases resident in England. All reported bloody diarrhoea, 10 with abdominal pain, seven with vomiting and five with fever. Eight of 12 cases required hospitalisation and four developed HUS. No deaths were reported.

Review of the ESQs of 11 cases yielded no obvious common exposure between cases. None reported foreign travel in the 7 days prior to onset. Three cases reported direct or indirect contact with ruminants but the wide geographical dispersal of cases suggested a nationally distributed foodborne source of infection was likely.

The case-case study identified that Retailer A was reported by 90.9% outbreak cases (10/11) compared to 19.8% of the control group (106/536, *P* < 0.001). No other supermarkets were reported more often amongst outbreak cases than the control group (Table 1). Among food exposures, 13 food categories were reported by more than half of outbreak cases with an OR >1 (Supplementary file S1). Free text analysis identified 20 items which were reported significantly more often amongst outbreak cases, among which consumption of beef from Retailer A was most frequently reported (Table 2).

**Table 1.** Frequency of shopping at national UK supermarket chains^a^ reported by outbreak cases compared to STEC- control cases by univariable analysis

Supermarket chain^a^	Cases (*n* = 11)			Control group (*n* = 537)^b^			Odds Ratio	95% CI	*P*-value
	Total	Exposed	%	Total	Exposed	%			
Retailer A	11	10	90.9	536	106	19.8	40.57	5.62–1764.05	<0.001
Retailer B	11	2	18.2	536	34	6.3	3.28	0.33–16.71	0.159
Retailer C	11	0	0.0	536	87	16.2	0	0.00–1.82	0.227
Retailer D	11	5	45.5	536	194	36.2	1.47	0.35–5.86	0.539
Retailer E	11	1	9.1	536	43	8.0	1.15	0.03–8.41	0.606
Retailer F	11	1	9.1	536	52	9.7	0.93	0.02–6.78	1.00
Retailer G	11	2	18.2	536	130	24.3	0.69	0.07–3.42	1.00
Retailer H	11	0	0.0	536	40	7.5	0	0.00–4.41	1.00
Retailer I	11	0	0.0	536	28	5.2	0	0.00–6.50	1.00

a Reporting of supermarket chains amongst cases was determined from parsing free text fields used to collect data on where specific food items were purchased into a new categorical variable.

b Control group comprised of all primary STEC O157:H7 cases, not associated with travel abroad or any known outbreaks. The controls were matched to cases on gender and age group and controls were only selected for the months September to December in 2017 and the preceding years (2009–2016).

**Table 2.** Frequency of food exposures^a^ among STEC outbreak cases compared to STEC-control cases by univariable analysis, NESSS data

Exposure^a^	Cases (*n* = 11)			Control group (*n* = 537)^a^			Odds ratio	95% CI	*P*-value
	Total	Exposed	%	Total	Exposed	%			
Beef from Retailer A	11	6	54.55	536	34	6.34	17.72	4.21–76.34	<0.001
Yoghurt from Retailer A	11	4	36.36	536	30	5.6	9.64	1.94–40.06	0.003
Other foods from Retailer D	11	4	36.36	536	37	6.9	7.71	1.57–31.72	0.006
Poultry from Retailer A	11	4	36.36	536	38	7.09	7.49	1.53–30.79	0.007
Rosemary	11	2	18.18	536	6	1.12	19.63	1.68–129.01	0.01
Vegetables from Retailer A	11	4	36.36	536	43	8.02	6.55	1.34–26.81	0.01
Pork sausages	11	4	36.36	536	45	8.4	6.23	1.28–25.48	0.012
Fruit from Retailer A	11	4	36.36	536	46	8.58	6.09	1.25–24.86	0.013
Crisps	11	2	18.18	536	8	1.49	14.67	1.32–88.72	0.015
Nuts	11	3	27.27	536	30	5.6	6.33	1.02–27.93	0.024
Pork from other shops	11	3	27.27	536	31	5.78	6.11	0.99–26.93	0.026
Other produce Retailer A	11	3	27.27	536	32	5.97	5.91	0.96–25.99	0.028
Other produce Retailer B	11	2	18.18	536	12	2.24	9.7	0.91–54.29	0.029
Chicken portions	11	6	54.55	536	129	24.07	3.79	0.94–15.91	0.031
Yoghurt Retailer D	11	4	36.36	536	64	11.94	4.21	0.88–17.05	0.037
Milk from Retailer D	11	5	45.45	536	99	18.47	3.68	0.87–14.74	0.04
Apple juice	11	4	36.36	536	67	12.5	4	0.83–16.17	0.042
Other produce from other shops	11	3	27.27	536	38	7.09	4.91	0.80–21.49	0.042
Poultry from Retailer B	11	2	18.18	536	16	2.99	7.22	0.70–38.93	0.047

a Exposures not collected via standard binary questions but coded through parsing free text fields into coded variables.

Seven cases were interviewed using a trawling questionnaire and all reconfirmed shopping at Retailer A. Consumption or handling of raw beef intended for cooking was reported by all seven and included one or more products of burgers (*n* = 7), minced beef (*n* = 3), beef pies (*n* = 2), steak (*n* = 1) and roast beef (*n* = 1). Six of seven cases reported purchasing the beef products from Retailer A.

Ten cases were re-interviewed between 8 December and 11 December 2017 using a questionnaire focussed on burger and mince products. Nine cases reported eating cooked frozen burgers from Retailer A and one from Retailer B. Left over raw product remained in three households and was retrieved and tested. Six cases provided loyalty card data.

Retailer A reviewed the purchase history for the six cases with loyalty card data. This confirmed that 4/6 had purchased the retailer's own branded frozen burgers between August and November 2017. Online shopping history from a further case (without a loyalty card) indicated that they had ordered a different type of Retailer A's own branded fresh burgers. From loyalty card data and patient interviews, specific product details were available for 10/12 cases. In total, nine reported burgers from Retailer A, seven a specific own-brand frozen burger product and two a specific own-brand fresh burger product.

### Food chain investigations

STEC O157:H7 was isolated from two leftover burgers sampled from homes of two different cases and reported as STEC O157:H7 PT2 Stx2 on 21 December 2017. On 29 December 2017, WGS confirmed that the two isolates were identical to the outbreak strain. A sample of burger retained at the meat preparation premises was tested and confirmed to have the outbreak strain by WGS.

Product trace-back investigations indicated the burgers were produced at a single meat preparation premise, who produced frozen burgers for all major UK supermarkets. Batch code and production information stamped on the product packages of the two samples obtained from cases freezers stated that they were produced on 5 September 2017 at 07:56 h. The third sample from the meat preparation premises from which the outbreak strain was confirmed by PHE was produced at 07:47 h on the same day.

All three burgers were likely to have been produced from the same batch of material. Fifteen different blocks of raw material from one supplier were used to produce the implicated product. A small quantity of the raw material from one of the batches was also used to produce burgers sold by Retailer B. (No outbreak cases were linked to burgers sold by Retailer B.) Four formal final burger products produced from the same batch of raw ingredient as the implicated batch but at different times were tested and no STEC was detected (Table 3). This indicated that the contamination may have been limited to one mix prepared between 07:00 and 08:00 h on 5 September 2017. The implicated burgers had a ‘best before’ date of September 2018. It was confirmed that the products were distributed exclusively across the UK and not exported abroad and that 30 252 packs had been produced from that batch.

**Table 3.** Microbiological sampling and STEC testing results of product samples taken during the outbreak investigation

Sample location	Sample type/description	Result
Meat preparation premises	Retailer A beef burger product produced on 5 September 2017, 06:02	STEC O157 not detected
Meat preparation premises	Retailer A beef burger product produced on 5 September 2017, 07.10	STEC O157 not detected
Outbreak case A's freezer	Retailer A beef burger product produced on 5 September 2017, 07:56	STEC O157 isolated: Confirmed as the outbreak strain
Outbreak case B's freezer	Retailer A beef burger product produced on 5 September 2017, 07:56	STEC O157 isolated: Confirmed as the outbreak strain
Outbreak case C's freezer	Retailer A beef burger product produced on 5 September 2017, 07:56	*stx* positive by PCR, STEC not isolated.
Meat preparation premises	Retailer A beef burger product produced on 5 September 2017, 07:47	STEC O157 isolated: confirmed as the outbreak strain
Meat preparation premises	Retailer A beef burger product produced on 5 September 2017, 11.50	STEC O157 not detected
Meat preparation premises	Retailer B beef burger product produced on 5 September 2017, 09.50	STEC O157 not detected

The meat preparation premises were inspected on 4 January 2018. A review of company records on cleaning, environmental swabbing and food product sampling results was undertaken. Fourteen hygiene swabs were taken at several points along the food processing areas at the premises. Two swabs showed *Enterobacteriaceae* counts of >10^2^ and <10^4^ cfu per swab but were negative for *β*-glucuronidase-positive *E. coli* and coagulase-positive *Staphylococci.* All 14 swabs were negative for STEC O157. Inspection of the premises confirmed that there were no breaches in compliance against food safety requirements.

The burger packs carried the advice ‘Check food is piping hot’ twice. The label had a safety statement with a ‘call out’ explanation mark on the side of the pack giving advice to cook ‘until no raw meat remains’. The cooking instructions had been verified by an independent laboratory who cooked the product under the grill using the cooking instructions as per the pack and concluded that the recommended temperature of 70 °C or above for 2 min would be achieved.

Retailer A undertook additional testing of the cooking instructions on 18 December 2017 (for grilling, the most common cooking method) which confirmed that post-cook, the burgers (including the core) remained at over 70 °C for at least 2 min. The FSA concluded that if the cooking instructions were adequately followed, the necessary temperature to achieve a 6-log reduction in *E. coli* in the burgers would be achieved and that the cooking guidance had an adequate safety margin, regardless of the method of cooking burgers (grilled or shallow fried).

Following confirmation of the vehicle of the outbreak, seven food handlers who prepared the burgers for eight outbreak cases were interviewed about storage and cooking of the burgers at home. All reported good storage and cooking practices at home, although this was self-reported. Four cases reported freezer temperatures between −20 and −25 °C, lower than the recommended freezer temperatures of around −18 °C.

### Outbreak control measures

Upon confirmation of STEC O157:H7 in two burger samples on 20 December 2017, Retailer A voluntarily removed the implicated products from shop shelves pending further investigations. Following confirmation of the STEC strain as PT2 on 21 December 2017, investigators considered all relevant factors including the severity of illness caused by the outbreak strain, the long shelf life (at least 12 months) of frozen burgers and the likelihood of customers having frozen burgers in their homes and recommended a voluntary recall of the implicated product. The FSA and Retailer A issued a product recall notice on the 22 December 2017 advising those who might have purchased any of the affected batches not to consume the product, and to return it to Retailer A for a full refund. Recall notices were displayed in store, media coverage publicised the recall and Retailer A directly contacted 57 000 customers who had purchased the products to notify them of the recall. A number of customers returned the product and approximately 19 000 of the 30 252 units were destroyed. Retailer B also removed frozen burgers made on two production runs from the same raw material on 22 December 2017.

No further cases linked to the outbreak have been reported to date, indicating that the intervention was successful.

## Discussion

This report summarises a geographically dispersed outbreak of STEC O157:H7 in the UK, where epidemiological and food sampling investigations led to the identification of frozen beef burgers sold by Retailer A as the vehicle of infection and product recall. Although the number of cases was relatively small, illness was severe with eight cases requiring hospitalisation, four of whom developed HUS. As the burgers were frozen and had a long shelf life, the product recall was particularly important. No further cases were detected, indicating that the intervention was successful in preventing additional cases or any remaining product was handled and cooked appropriately.

The initial detection of the outbreak in mid-November, when there were just four cases, was facilitated through the use of routine WGS of all clinical STEC isolates in England, which has improved the ability to detect relatively small, geographically dispersed outbreaks, particularly when cases are more widespread in time, as seen in this outbreak with cases reported over several weeks. The value in collecting routinely collected standardised exposure data on all STEC O157 cases to inform hypothesis generation and outbreak investigation was demonstrated here and has had proven utility in previous outbreaks in the UK [9, 18].

Reviewing shopping loyalty card data in this outbreak was also particularly useful to identify the exact product purchased by cases. The benefits of using loyalty cards have been demonstrated in investigations of foodborne outbreaks elsewhere [19]. In the UK, they have not previously been used and realising their benefit relies on cases being willing to share their loyalty card data and the co-operation and support of the retailers in analysing their dataset for shopping history. In suspected foodborne outbreak investigations, we recommend collecting supermarket loyalty card data from cases with appropriate consent for further analysis of their shopping history through the retailer(s). It is important to acknowledge that loyalty card data can be complete only if customers present the card during every shopping event at the retailer, which is unlikely to happen in practice. Furthermore, a record of purchase of a specific product in the retailer database does not necessarily mean that cases were definitely exposed to the product.

Early outbreaks of STEC in the USA were linked to hamburger consumption, leading to STEC being nicknamed as the ‘burger bug’ [20]. Outbreaks associated with ground beef and burgers have persistently recurred in the USA since that time [12]. As cattle are well-known carriers of STEC, surface contamination of raw beef with STEC is a recognised risk. The mincing process required for burgers is likely to spread STEC throughout the burger, thereby necessitating a cooking process that can destroy STEC. Due to variation in cooking practices and performance of cooking appliances, and failure to follow cooking instructions or avoid cross-contamination in the home, we suspect that sporadic STEC cases linked to frozen burgers cooked at home are underestimated.

The prominence of burgers and ground beef as a causative vehicle in STEC outbreaks in North America has not been mirrored in the UK. Large outbreaks associated with butcher's premises have occurred in the past, particularly a large central Scotland outbreak in 1996 and another in South Wales in 2005 [21, 22]. However, in the following years, interventions were implemented across the UK aimed at reducing the risk of infection in catering, retail and meat hygiene sectors. They appeared to be effective as there was a decline in outbreaks caused by cross-contaminated cooked meats [5], and are likely to have reduced the risks for contamination from carcasses upstream of production for ground beef and burger products.

In a recent study, 21% and 24% of cattle farms surveyed in Scotland, and England and Wales, respectively, were positive for STEC O157 [23], while between 0.3% and 1.1% of raw beef products sampled in the UK have previously been reported to be contaminated with STEC O157 [24–26]. This also suggests that control measures upstream of cooking are largely effective, but contamination can persist. The trend to deliberately undercook burgers presents an additional risk therefore. However, only four small, local outbreaks associated with consumption of burgers occurred in England and Wales between 2009 and 2015 and these were reported as likely as due to consumption of undercooked fresh burgers or cross-contamination outside of the home (unpublished, in-house data). This is the first report of a national outbreak of STEC associated with burger consumption, and indeed the first known outbreak in the UK linked to frozen burgers. In the literature, frozen burgers were identified as the vehicle in three previous STEC outbreaks [27–29], including a large community-wide outbreak of 69 cases in France in 2005 [28]. The investigations suggested that heavy contamination of a specific batch caused the outbreak, but the precise source of that contamination was not known. Although the authors suggest the transfer of STEC from significantly contaminated carcasses to meat during slaughter and processing, they also state that at that time, beef burgers were commonly consumed under-cooked in France.

As STEC outbreaks associated with frozen burgers are very rare, consideration was given to why this outbreak occurred and/or was detected. The outbreak strain had the Shiga toxin subtype stx2a which is significantly associated with severe disease. The heightened disease severity would have increased the likelihood of cases seeking diagnosis thereby facilitating detection and investigation of the outbreak. However, STEC O157:H7 harbouring stx2a are common in the UK and the virulence profile of the outbreak strain alone does not explain this outbreak.

Investigators considered other potential contributory factors, such as inadequate cooking and possible failures in food safety processes during the production of this specific batch. Investigations at the approved meat preparation premises by the LA did not identify any failures in food safety processes, or any breaches in food hygiene regulations.

In terms of control measures, given that STEC can be present in raw beef, adequate controls during the production, storage and cooking of burgers are essential to minimise the risk of illness. Among these, adequate cooking is the critical control point. Current advice is that burgers should be cooked to remain at 70 °C for 2 min, and adequate labelling is important for informing consumers on safe cooking of burgers [30]. Experts at the FSA carefully reviewed the guidance and evidence on cooking instructions provided on the burger pack and were satisfied that the instructions were adequate with a safety margin. Interviews of the food handlers who prepared the burgers at home revealed good storage and cooking practices overall and there was no clear evidence for cross-contamination during the cooking process or consumption of burgers eaten undercooked. Nevertheless, these behaviours were self-reported, and cases were infected from consuming the contaminated burgers, so the potential for cross-contamination during storage and cooking at home or inadequate cooking of the burgers are risk factors that may have been contributing factors in the foodborne transmission event.

Detection of this outbreak when there were four cases, demonstrates the value of WGS in early detection of geographically dispersed outbreaks when relatively small numbers of people are affected. The investigations highlight the need to maintain robust surveillance and rapid outbreak response procedures with access to advanced epidemiological and microbiological tools, methods and expertise. The effective multi-agency response, with vital contributions from various divisions of PHE, FSA, the LA and Scottish authorities, was critical in the investigation and management of this outbreak.

## Data Availability

The data that support the findings of this study are openly available in Supplementary file S1.

## Supplementary material

For supplementary material accompanying this paper visit http://dx.doi.org/10.1017/S0950268820001582. click here to view supplementary material
