# Supplementary material for: Investigation into a national outbreak of STEC O157:H7 associated with frozen beef burgers, UK, 2017
Source: Epidemiol Infect. 2020 Jul 16;148:e215. doi: 10.1017/S0950268820001582 (PMC7522850; doi:10.1017/S0950268820001582)
Supplement: Supplementary file 1 [file S0950268820001582sup001.docx]

**Epidemiology and Infection.**

**Investigation into a national outbreak of STEC O157:H7 associated with frozen beef burgers, UK, 2017**

Lisa Byrne, Lukeki Kaindama, Maria Bentley, Claire Jenkins, Heather Aird, Isabel Oliver and Karthik Paranthaman.

**Supplementary table (S1).**

Table S1. Frequency of food exposures reported by STEC outbreak cases compared to STEC-control cases by univariable analysis, NESSS data

| **Exposure*** | **Cases(n=11)** | | **Control group (n=537)** | | **Odds Ratio** | **95% CI** | **p-value** |
| --- | --- | --- | --- | --- | --- | --- | --- |
|  | **Total** | **%** | **Total** | **%** |  |  |  |
| Cooked poultry | 10 | 90.9 | 354 | 65.9 | 5.14 | 0.72 - 224.3 | 0.084 |
| Pasteurised milk | 10 | 90.9 | 384 | 71.5 | 3.96 | 0.55 - 172.9 | 0.159 |
| Eating outside of home | 9 | 81.8 | 390 | 72.6 | 1.68 | 0.34 - 16.2 | 0.503 |
| Hard cheese | 9 | 81.8 | 312 | 58.1 | 3.23 | 0.66 - 30.92 | 0.115 |
| Cooked beef | 8 | 72.7 | 263 | 49.0 | 2.80 | 0.65 - 16.4 | 0.120 |
| Cooked pork | 8 | 72.7 | 158 | 29.4 | 6.40 | 1.5 - 37.7 | 0.002 |
| Soft fruit/berries | 8 | 72.7 | 126 | 23.5 | 8.80 | 2.03 - 51.3 | 0.000 |
| Raw fruit | 8 | 72.7 | 337 | 62.8 | 1.57 | 0.37 - 9.31 | 0.503 |
| Juice | 8 | 72.7 | 247 | 46.0 | 3.12 | 0.74 - 18.4 | 0.080 |
| Other foods | 8 | 72.7 | 212 | 39.5 | 4.10 | 0.96 - 24.05 | 0.026 |
| Raw vegetables | 7 | 63.6 | 217 | 40.4 | 2.57 | 0.64 - 12.11 | 0.122 |
| Ice cream | 7 | 63.6 | 89 | 16.6 | 8.79 | 2.17 - 41.57 | <0.001 |
| Yoghurt | 6 | 54.5 | 260 | 48.4 | 1.30 | 0.319 - 5.34 | 0.692 |
| Processed meats | 5 | 45.5 | 130 | 24.2 | 2.60 | 0.62 - 10.4 | 0.106 |
| Raw beef | 3 | 27.3 | 80 | 14.9 | 2.13 | 0.36 - 9.12 | 0.259 |
| Raw meat | 3 | 27.3 | 140 | 26.1 | 1.06 | 0.179 - 4.5 | 0.913 |
| Cured meats | 3 | 27.3 | 145 | 27.0 | 1.01 | 0.17 - 4.3 | 0.987 |
| Fish | 3 | 27.3 | 240 | 44.7 | 0.46 | 0.08 - 1.95 | 0.248 |
| Soft cheese | 3 | 27.3 | 107 | 19.9 | 1.50 | 0.25 - 6.4 | 0.550 |
| Pre-cut fruits | 3 | 27.3 | 24 | 4.5 | 8.00 | 1.3 - 35.8 | 0.001 |
| Pet/animal feed | 3 | 27.3 | 56 | 10.4 | 3.20 | 0.53 - 13.85 | 0.075 |
| Raw poultry | 2 | 18.2 | 75 | 14.0 | 1.36 | 0.14 - 6.8 | 0.692 |
| Pre-packaged salad | 2 | 18.2 | 99 | 18.4 | 0.98 | 0.1 - 4.85 | 0.980 |
| Other salad | 2 | 18.2 | 200 | 37.2 | 0.37 | 0.039 - 1.83 | 0.193 |
| Herbs | 2 | 18.2 | 90 | 16.8 | 1.10 | 0.114 - 5.44 | 0.903 |
| Other raw meat | 1 | 9.1 | 18 | 3.4 | 2.90 | 0.06 - 22.2 | 0.302 |
| Other cooked meat | 1 | 9.1 | 86 | 16.0 | 0.52 | 0.012 - 3.77 | 0.535 |
| Shellfish | 1 | 9.1 | 46 | 8.6 | 1.06 | 0.025 - 7.79 | 0.950 |
| Cream | 1 | 9.1 | 83 | 15.5 | 0.54 | 0.012 - 3.93 | 0.560 |
| sprouted seeds | 1 | 9.1 | 17 | 3.2 | 3.05 | 0.067 - 23.7 | 0.28 |

_* Binary response variables as collected on the national enhanced surveillance questionnaire_
